# Safety and efficacy of PRaG therapy in elderly cancer patients

**DOI:** 10.1093/immadv/ltag019

**Published:** 2026-08-11

**Authors:** Xiangrong Zhao, MengMeng Yang, Junjun Zhang, Yuehong Kong, Meiling Xu, Rongzheng Chen, Qian Yin, Pengfei Xing, Liyuan Zhang

**Affiliations:** Center of PRaG Therapy, The Second Affiliated Hospital of Soochow University, Suzhou, China; Laboratory for Combined Radiotherapy and Immunotherapy of Cancer, The Second Afﬁliated Hospital of Soochow University, Suzhou, China; People’s Hospital of Ningxia Hui Autonomous Region, Ningxia Medical University, Ningxia, China; Center of PRaG Therapy, The Second Affiliated Hospital of Soochow University, Suzhou, China; Laboratory for Combined Radiotherapy and Immunotherapy of Cancer, The Second Afﬁliated Hospital of Soochow University, Suzhou, China; Center of PRaG Therapy, The Second Affiliated Hospital of Soochow University, Suzhou, China; Laboratory for Combined Radiotherapy and Immunotherapy of Cancer, The Second Afﬁliated Hospital of Soochow University, Suzhou, China; Center of PRaG Therapy, The Second Affiliated Hospital of Soochow University, Suzhou, China; Laboratory for Combined Radiotherapy and Immunotherapy of Cancer, The Second Afﬁliated Hospital of Soochow University, Suzhou, China; Center of PRaG Therapy, The Second Affiliated Hospital of Soochow University, Suzhou, China; Laboratory for Combined Radiotherapy and Immunotherapy of Cancer, The Second Afﬁliated Hospital of Soochow University, Suzhou, China; Center of PRaG Therapy, The Second Affiliated Hospital of Soochow University, Suzhou, China; Laboratory for Combined Radiotherapy and Immunotherapy of Cancer, The Second Afﬁliated Hospital of Soochow University, Suzhou, China; Center of PRaG Therapy, The Second Affiliated Hospital of Soochow University, Suzhou, China; Laboratory for Combined Radiotherapy and Immunotherapy of Cancer, The Second Afﬁliated Hospital of Soochow University, Suzhou, China; Center of PRaG Therapy, The Second Affiliated Hospital of Soochow University, Suzhou, China; Laboratory for Combined Radiotherapy and Immunotherapy of Cancer, The Second Afﬁliated Hospital of Soochow University, Suzhou, China

**Keywords:** PD-1/PD-L1 inhibitors, elderly cancer, radiotherapy, immunotherapy, PRaG therapy

## Abstract

**Introduction:**

PD-1/PD-L1 inhibitors lack evidence-based support in patients aged ≥75 years due to their frequent exclusion from clinical trials. This study aimed to evaluate the safety and efficacy of PRaG therapy—combining PD-1/PD-L1 inhibitors with radiotherapy—specifically in this elderly demographic.

**Materials and methods:**

Data were collected from patients aged ≥75 years who received PRaG therapy at our center between 1 January 2019, and 31 December 2024. Eligible patients were required to have at least one post-baseline efficacy evaluation. Tumor response was evaluated according to RECIST v1.1, and adverse events were graded using CTCAE v5.0.

**Results:**

A total of 36 elderly patients aged ≥75 years were included, with a median age of 77 years (range: 75–93). The objective response rate (ORR) was 23.5%, and the disease control rate (DCR) was 70.6%. After a median follow-up of 46.3 months, the median progression-free survival (mPFS) was 7.0 months (95% CI: 2.4–11.6 months) and the median overall survival (mOS) was 11.1 months (95% CI: 5.3–16.9 months). The Geriatric Nutritional Risk Index (GNRI) was associated with the efficacy of PRaG therapy, as well as PFS and OS. Any-grade treatment-related adverse events (TRAEs) occurred in 34 patients (94.4%), with 3 patients (8.3%) experiencing ≥Grade 3 TRAEs. No Grade 5 TRAEs were observed.

**Conclusion:**

The PRaG regimen appears feasible and tolerable in selected elderly patients with advanced cancer. Given the retrospective nature of this small cohort, an ongoing prospective trial (NCT06112041) will further validate these findings. Combining PRaG with robust nutritional support represents a rational hypothesis for future investigation.

## Introduction

Although PD-1/PD-L1 inhibitors have revolutionized cancer therapy, there is currently limited prospective evidence for their optimal use in patients aged ≥75 years, primarily because this vulnerable population is frequently underrepresented in pivotal clinical trials. With the progressive ageing of the global population and the continuously rising incidence of cancer, the clinical needs of this patient demographic are expanding. Key areas of research focus on combining PD-1/PD-L1 inhibitors with chemotherapy, molecular targeted therapies, and radiotherapy. PRaG therapy, a combination regimen based on a PD-1/PD-L1 inhibitor, radiotherapy, and GM-CSF, is utilized as a salvage treatment for refractory advanced tumors. The acronym ‘PRaG’ represents these three core components. The essence of PRaG therapy lies in the use of ionizing radiation to expose tumor antigens, thereby generating an *in situ* ‘tumor vaccine effect.’ In this context, the PD-1 inhibitor and GM-CSF function as vaccine adjuvants, promoting the proliferation of antigen-presenting cells and enhancing the immune response efficacy of the tumor vaccine. This approach has benefited thousands of patients in China and has been adopted by over one hundred medical institutions. Preliminary results from PRaG therapy clinical studies (ChiCTR1900026175, NCT04892498, NCT05115500, NCT05435768, NCT05501340, NCT05603013, NCT05530200, NCT06345599, NCT06112041) indicate that PRaG therapy is an effective and safe salvage therapy for patients with advanced refractory solid tumors.

The PRaG 1.0 study evaluated the efficacy and safety of a triple combination regimen consisting of a PD-1/PD-L1 inhibitor, radiotherapy, and GM-CSF in patients with refractory advanced tumors. In the published PRaG 1.0 study, which enrolled patients with advanced tumors who had failed prior anticancer treatments (including 7 patients aged ≥75 years), the ORR was 16.7%, the DCR was 46.3%, the mPFS was 4.0 months (95% CI, 3.3–4.8 months), and the mOS was 10.5 months (95% CI, 8.7–12.2 months) [1]. The PRaG regimen has yielded promising preliminary results at our center in treating various advanced refractory malignancies, including esophageal [2], gastric [3], colon [4], pancreatic [5], lung [6], breast [7], and nasopharyngeal cancers [8]. Moreover, according to published literature, other institutions applying the PRaG regimen have reported positive antitumor effects in refractory gastric cancer [9], ovarian cancer [10], uterine leiomyosarcoma [11], malignant perivascular epithelioid cell tumor [12], and thyroid cancer [13]. Consequently, this technology was listed by the National Health Commission of China as a national health promotion technology in 2024 (No. RKJS-20240234).

PRaG therapy has been routinely implemented as a clinical technology at our center since 2022 (JD-LJ-2022–001-01). In this study, we analyzed the outcomes of elderly patients (≥75 years) who had previously received the PRaG therapy regimen at our institution to assess its safety and efficacy and to identify factors associated with treatment response, aiming to provide a new therapeutic option for this patient population.

## Materials and methods

This study was approved by the Ethics Committee of The Second Affiliated Hospital of Soochow University (JD-HG-2025-039). Clinical data of patients aged ≥75 years, who received PRaG therapy between 1 January 2019, and 31 December 2024, were collected. The PRaG regimen consisted of hypofractionated radiotherapy (5–8 Gy/fraction for 2–3 fractions) targeting selected primary or metastatic lesions, concurrent daily subcutaneous GM-CSF (200 μg) for 5–14 days starting on the first day of radiotherapy, and the administration of a PD-1/PD-L1 inhibitor within one week post-radiotherapy. This treatment cycle was repeated every 3 weeks, with imaging evaluations performed every 6 weeks. All included patients had completed baseline examinations and at least one radiological efficacy assessment. The follow-up cutoff date was 1 March 2025. The following data were collected: patient gender, height, weight, age, Eastern Cooperative Oncology Group (ECOG) performance status, primary tumor location, Age-Adjusted Charlson Comorbidity Index (aCCI), and baseline concurrent medications (specifically focusing on systemic corticosteroids, defined as receiving a prednisone equivalent of ≥≥ 10 mg/day at treatment initiation).

Treatment efficacy was assessed using RECIST v1.1 [14] based on baseline and on-treatment CT, MRI, Positron Emission Tomography-Computed Tomography (PET-CT), and Emission Computed Tomography (ECT) scans. Adverse events were evaluated according to the Common Terminology Criteria for Adverse Events (CTCAE) v5.0 [15]. The primary endpoint was the objective response rate (ORR). Secondary endpoints included toxicity, disease control rate (DCR), progression-free survival (PFS), and overall survival (OS). The median follow-up time was calculated using the standard reverse Kaplan-Meier method. For the analysis of PFS, patients who initiated a subsequent line of anticancer therapy prior to documented disease progression were censored at the date of their last evaluable tumor assessment. For overall survival (OS), survival time was calculated regardless of subsequent therapies, with death from any cause counted as an event.

Lymphocyte subsets (CD3+, CD4+, and CD8+T cells) were enumerated using multi-color flow cytometry. Briefly, whole peripheral blood samples were stained with a standard clinical antibody panel, including FITC-conjugated anti-CD3, APC-conjugated anti-CD4, and PE-conjugated anti-CD8 antibodies. Data acquisition was performed on a clinical flow cytometer (e.g. BD FACSCanto II). The gating strategy involved initial gating on the lymphocyte population based on forward and side scatter (FSC/SSC) characteristics, followed by the identification of CD3+ total T cells, and subsequent sub-gating to quantify CD3+CD4+ (helper T cells) and CD3+CD8+ (cytotoxic T cells) populations. Absolute cell counts (cells/μL) were calculated using a standard single- or dual-platform method established by our institutional clinical laboratory.

Statistical analysis was performed using SPSS 26.0 software. The Shapiro-Wilk test (*α* = 0.05) was used to assess normality. Normally distributed data were described as mean ± standard deviation (mean ± SD), and differences between groups were analyzed using the *t*-test. Non-normally distributed variables were described using the median and interquartile range [M (P25, P75)], and group differences were compared using non-parametric tests. Categorical variables were presented as *n* (%) and analyzed using the chi-square test. Survival curves were generated using the Kaplan-Meier method. Univariate and multivariate Cox proportional hazards models were used to analyze the association of baseline characteristics with PFS and OS to identify independent prognostic factors. A *P*-value < 0.05 was considered statistically significant.

## Results

A total of 36 patients were included in this study, of whom 69.4% (25/36) were male and 30.6% (11/36) were female. The median age was 77 years (range: 75–93). A total of 44.4% (16/36) of patients had received ≥2 prior lines of systemic therapy, while 25.0% (9/36) were treatment-naïve. The cohort included a diverse patient population with various primary sites: colorectal cancer in 10 cases (27.8%), esophageal cancer in 4 (11.1%), lung cancer in 4 (11.1%), gynecological tumors in 4 (11.1%), pancreatic cancer in 3 (8.3%), gastric cancer in 2 (5.5%), renal cancer in 2 (5.5%), head and neck tumors in 2 (5.5%), and other rare cancers in 5 (13.9%). Prior to PRaG therapy, 75% (27/36) of patients had an ECOG performance status of ≥2. Liver metastases were present in 27.8% (10/36) of patients, and 44.4% (16/36) had distant lymph node metastases. Geriatric-specific assessments revealed a severe comorbidity burden expected in this population, with a median modified age-adjusted CCI of 10 (range: 9–13). Notably, 30 patients (83.3%) had a CCI score of ≥10. Detailed baseline characteristics are presented in Table 1.

**Table 1:** Patient characteristics.

Characteristic	No. (%)
**Age (years)**	
75–79	27 (75.0%)
≥80	9 (25.0%)
Median	77
**Gender**	
Male	25 (69.4%)
Female	11 (30.6%)
**Charlson Comorbidity Index (CCI)**	
**≥10**	30 (83.3%)
**<10**	6 (16.7%)
**Primary cancer sites**	
Colorectum	10 (27.8%)
Esophagus	4 (11.1%)
Lung	4 (11.1%)
Ovary	4 (11.1%)
Pancreas	3 (8.3%)
Stomach	2 (5.5%)
Kidney	2 (5.5%)
Head and neck	2 (5.5%)
Others	5 (13.9%)
**Metastatic organs involved**	
0–2	26 (72.2%)
≥3	10 (27.8%)
**No. of prior systemic therapies**	
0	9 (25.0%)
1	11 (30.6%)
≥2	16 (44.4%)
**ECOG performance status**	
0–1	9 (25.0%)
≥2	27 (75.0%)
**PD-L1**	
≥1%	4 (11.1%)
<1%	7 (19.4%)
Unknown	25 (69.5%)
**Liver metastasis**	
Yes	10 (27.8%)
No	26 (72.2%)
**Lymphatic metastasis**	
Yes	16 (44.4%)
No	20 (55.6%)

### Efficacy assessment and factor analysis

Of the 36 patients, two were not evaluable for efficacy. Among the 34 evaluable patients, response assessment based on RECISTv1.1 criteria revealed CR in 2 patients (5.6%), PR in 6 (16.7%), SD in 16 (44.4%), and PD in 10 (27.8%). For the evaluable cohort, the ORR was 23.5%, and the DCR was 70.6%. Comparison of characteristics between the disease control (CR + PR + SD) and progressive disease (PD) groups demonstrated that the number of prior systemic therapies, ECOG performance status, presence of liver metastases, Geriatric Nutritional Risk Index (GNRI), and baseline serum albumin were significantly associated with treatment efficacy. No statistically significant differences were found for age, presence of lymph node metastases, BMI, or absolute counts of lymphocyte subsets before and after treatment. Details are provided in Table 2.

**Table 2:** Comparison of characteristics between complete response (CR)+ partial response (PR)+ stable disease (SD), and progressive disease (PD) groups after treatment.

Characteristics	PR + CR + SD (*n* = 24)	PD (*n* = 10)	*P* value
Age, year	79.00 ± 4.40	77.30 ± 1.95	0.252
**ECOG score, *n* (%)**			**0**.**035**
0–1	8 (33.3%)	0 (0.0%)	
≥2	16 (66.7%)	10 (100.0%)	
Liver metastases, *n* (%)	4 (16.7%)	6 (60.0%)	**0**.**034**
Lymph nodes metastases, *n* (%)	11 (45.8%)	5 (50.0%)	0.824
**Baseline**			
BMI	22.65 ± 3.57	20.55 ± 2.50	0.102
Number of previous systemic therapy	1.00 (0.00–2.00)	2.00 (1.00–3.30)	**0**.**007**
GNRI	98.56 ± 8.57	92.07 ± 6.18	**0**.**038**
CD4+/CD8+ (%)	1.42 (1.10–2.10)	1.23 (1.00–1.70)	0.484
Baseline CD3+ T cells (cells/μL)	646.51 (556.34–937.86)	730.50 (484.58–1239.52)	0.584
Baseline CD4+ T cells (cells/μL)	445.33 ± 220.02	478.70 ± 317.57	0.727
Baseline CD8+ T cells (cells/μL)	317.63 ± 264.31	361.40 ± 206.31	0.644
Baseline B cells (cells/μL)	80.00 (54.80–127.00)	74.500 (21.00–269.00)	0.762
Baseline NK cells (cells/μL)	235.00 (159.80–376.50)	216.00 (59.80–335.30)	0.623
Treg	8.10 (5.70–11.70)	5.90 (4.90–18.80)	0.464
Albumin	39.71 ± 4.35	36.50 ± 3.31	**0**.**045**
**After 2 treatment cycles**			
ΔCD4+/CD8+%	2.03 ± 1.89	1.40 ± 0.23	0.318
ΔCD3+ T cells	1.00 (0.80–1.20)	0.85 (0.60–1.10)	0.265
ΔCD4+ T cells	0.90 (0.80–1.20)	0.94 (0.60–1.30)	0.985
ΔCD8+ T cells	1.03 (0.70–1.50)	0.80 (0.50–1.30)	0.290
ΔB cells	0.46 (0.30–0.70)	0.38 (0.20–0.70)	0.461
ΔNK cells	0.93 (0.60–1.10)	0.91 (0.50–1.40)	0.705

Δ values represent the ratio of cell counts after 2 cycles of treatment to baseline counts.

Among the 36 included patients, all completed at least two cycles of PRaG therapy. Two patients (5.6%) were unevaluable for tumor response, as they did not return to our institution for scheduled radiological assessment following treatment; notably, neither patient experienced early clinical deterioration or death during the treatment course. Survival outcomes for these two patients were obtained via telephone follow-up and were included in the PFS and OS analyses.

### Survival outcomes and factor analysis

**Figure 1:**
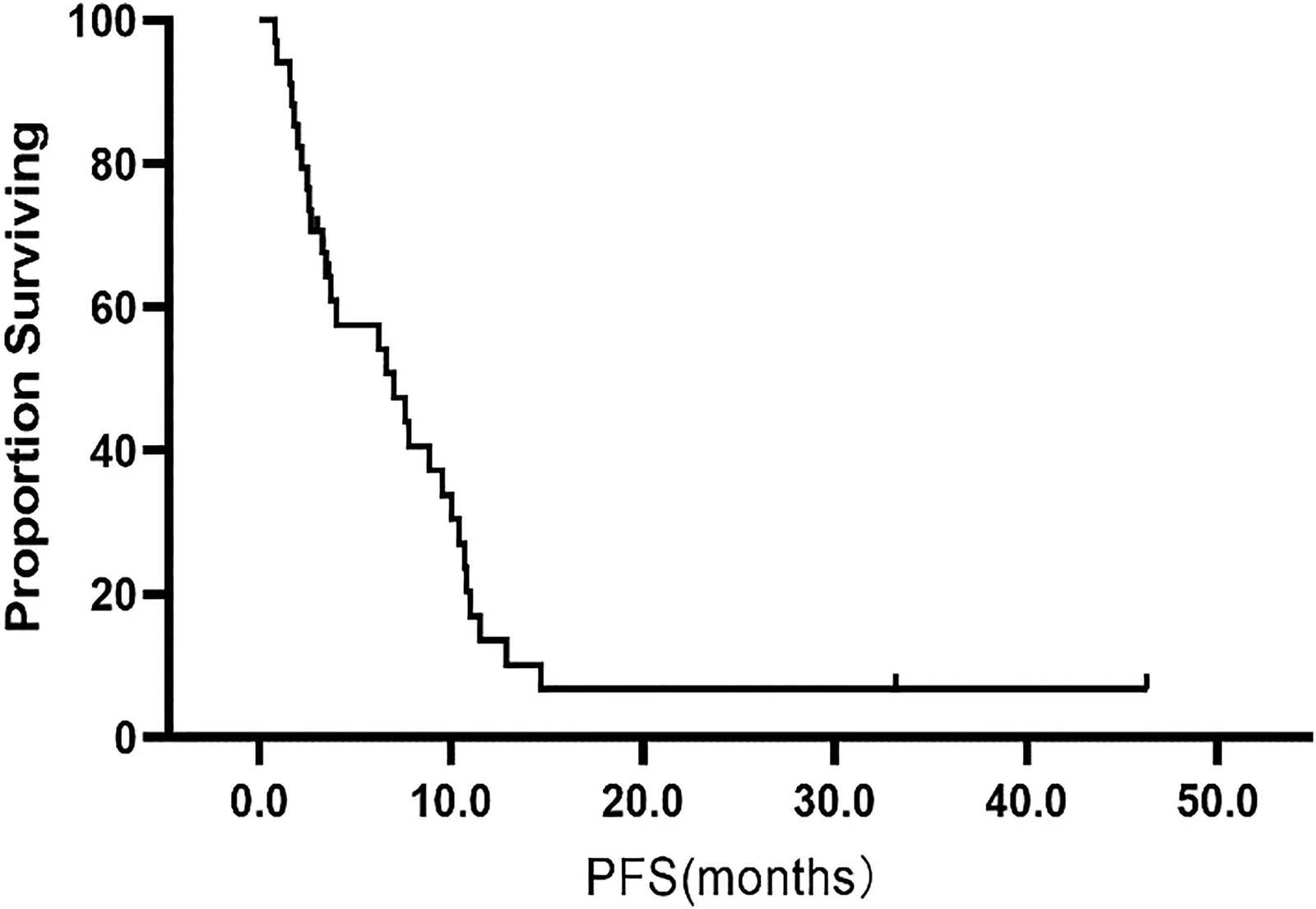
Kaplan-Meier curve of PFS for elderly patients receiving PRaG therapy.

**Figure 2:**
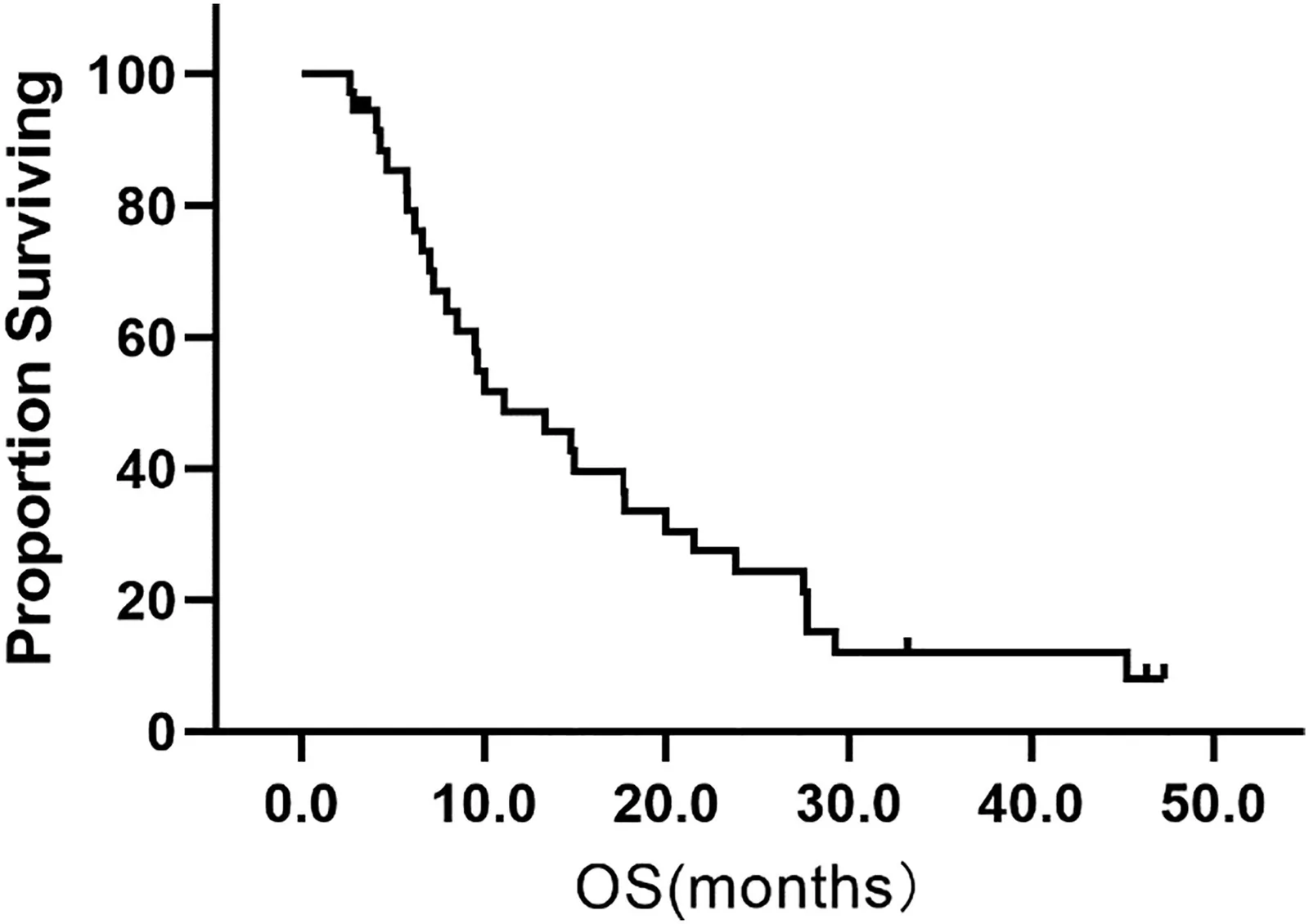
Kaplan-Meier curve of OS for elderly patients receiving PRaG therapy.

As of 1 March 2025, the median follow-up time was 46.3 months. The median progression-free survival (mPFS) was 7.0 months (95% CI, 2.4–11.6 months), and the median overall survival (mOS) was 11.1 months (95% CI, 5.3–16.9 months). Survival curves are shown in Figs 1 and 2.

To further investigate the impact of prior treatments, an exploratory subgroup analysis was conducted comparing treatment-naïve and pretreated patients. The treatment-naïve subgroup demonstrated numerically superior outcomes compared with the pretreated subgroup, with an ORR of 33.3% versus 20.0%. Similarly, the treatment-naïve group exhibited a longer median PFS [8.93 months (95% CI: 5.56–12.30) vs. 3.67 months (95% CI: 2.60–4.75); *P* = 0.225] and median OS [17.73 months (95% CI: 4.79–30.67) vs. 9.63 months (95% CI: 5.80–13.46); *P* = 0.435]. Although these differences did not reach statistical significance, likely due to the limited sample size of the subgroup, a clear trend favoring earlier intervention was observed.

Univariate analysis for PFS revealed that the GNRI, presence of liver metastases, baseline serum albumin, baseline hemoglobin, and the change in B lymphocyte percentage (ΔB cell%) were associated with PFS. In the multivariate Cox regression analysis of these variables, presented in Table 3, the GNRI (HR = 0.87, 95% CI: 0.15–0.87, *P* = 0.023), presence of liver metastases (HR = 2.78, 95% CI: 1.03–7.48, *P* = 0.043) and baseline hemoglobin (HR = 0.96, 95% CI: 0.93–0.99, *P* = 0.001) were identified as independent predictors of PFS.

**Table 3:** Multivariate analysis of PFS.

Variable	HR	95% CI	*P* value
Baseline albumin	1.26	0.96–1.65	0.102
Baseline hemoglobin	0.96	0.93–0.99	0.001
ΔB cell (%)	1.49	0.26–8.49	0.653
GNRI	0.36	0.15–0.87	0.023
Liver metastases	2.78	1.03–7.48	0.043

Δ value represents the ratio of cell counts after 2 cycles of treatment to baseline counts.

Univariate analysis for OS indicated that the GNRI, baseline serum albumin, baseline hemoglobin, baseline neutrophil-to-CD3+ T lymphocyte ratio, baseline neutrophil-to-CD4+ T lymphocyte ratio, and baseline neutrophil-to-CD8+ T lymphocyte ratio were associated with OS. In the multivariate Cox regression analysis, shown in Table 4, the GNRI (HR = 0.91, 95% CI: 0.85–0.96, *P* = 0.002) and baseline neutrophil-CD8+ T lymphocyte Ratio (HR = 0.99, 95% CI: 0.99–1.01, *P* = 0.802) were identified as independent predictors of OS.

**Table 4:** Multivariate analysis of OS.

Variable	HR	95% CI	*P* value
Baseline albumin	1.23	0.96–1.60	0.105
Baseline hemoglobin	0.97	0.94–0.99	0.010
Baseline neutrophil-CD3+ T lymphocyte Ratio	1.13	0.96–1.32	0.126
Baseline neutrophil-CD4+ T lymphocyte Ratio	0.99	0.96–1.01	0.234
Baseline neutrophil-CD8+ T lymphocyte Ratio	0.95	0.89–0.99	0.044
GNRI	0.91	0.85–0.96	0.002
Baseline neutrophil-CD8+ T lymphocyte Ratio	0.99	0.99–1.01	0.802

### Safety analysis

All 36 patients who received PRaG therapy were included in the safety analysis. A total of 34 patients (94.4%) experienced treatment-related adverse events (TRAEs) of any grade. The most common TRAEs included fatigue (75.0%), fever (55.6%), anorexia (36.1%), rash (16.7%), hypoxemia (13.9%), thyroid dysfunction (11.1%), pneumonia (8.3%), vomiting (5.6%), arrhythmia (2.8%), and myocarditis (2.8%). No grade 5 TRAEs were observed during the study. Three patients (8.3%) experienced grade ≥3 TRAEs, leading to treatment discontinuation: one case of immune-related myocarditis, one of immune-related dermatitis, and one of severe pneumonia. Details are provided in Table 5.

**Table 5:** Treatment-related adverse events (TRAEs).

Parameter	G1-2	≥G3
Fatigue	27 (75.0%)	0
Fever	20 (55.6%)	0
Anorexia	13 (36.1%)	0
Rash	6 (16.7%)	1 (2.8%)
Decrease in pulse oxygen saturation^a^	5 (13.9%)	0
Hypothyroidism	4 (11.1%)	0
Immune-related pneumonitis	2 (5.6%)	0
Radiation pneumonitis	0	1 (2.8%)
Vomiting	2 (5.6%)	0
Arrhythmia	1 (2.8%)	0
Myocarditis	0	1 (2.8%)

^a^The episodes of decreased pulse oxygen saturation were identified as adverse reactions associated with the administration of GM-CSF. These were typically transient and likely related to GM-CSF-induced mild capillary leak syndrome or systemic inflammatory responses, which resolved with standard supportive oxygen therapy.

Regarding the grade ≥3 adverse events, brief case-level details are as follows:

1. Immune-related myocarditis (*n* = 1): The patient was clinically asymptomatic without obvious chest tightness or discomfort, presenting primarily with a significant elevation in cardiac troponin levels. Following prompt intervention with high-dose systemic corticosteroids (methylprednisolone), the troponin levels gradually declined and completely normalized.
2. Severe pneumonitis (radiation-induced, *n* = 1): The patient presented with clinically significant chest tightness and dyspnea (shortness of breath). The condition was managed with systemic corticosteroid therapy, which led to a substantial clinical improvement and relief of respiratory symptoms.
3. Immune-related dermatitis (*n* = 1): This severe cutaneous toxicity was actively managed with a combination regimen of high-dose systemic corticosteroids (methylprednisolone) and antihistamines. Under this treatment, the patient’s symptoms gradually improved and eventually resolved.

## Discussion

As the global population ages, the number of elderly patients with malignant tumors is steadily increasing. Despite significant advances in cancer therapy over the past decades, age remains a major barrier to participation in clinical trials [16]. In trials registered with the U.S. Food and Drug Administration (FDA), only 24% of participants are aged ≥70 years [17], whereas patients over 75 constitute nearly 10% of the total cancer patient population [18]. Consequently, the optimal treatment strategies for elderly patients remain ill-defined. Furthermore, elderly patients generally exhibit poorer tolerance to chemotherapy [19], leading to a lower proportion of this demographic receiving guideline-recommended treatments.

With the advent of immunotherapy, immune checkpoint inhibitors (ICIs) have proven to be effective agents for a multitude of malignancies [20]. Previous studies indicate that immunotherapy provides durable antitumor responses and high 5-year survival rates across various tumor types. Compared with chemotherapy, immunotherapy is associated with a lower incidence of toxic reactions and lacks delayed myelosuppression or cumulative toxicity [21, 22]. A retrospective study exploring the efficacy of ICIs in patients with advanced non-small cell lung cancer (NSCLC) across different age groups reported that the mOS benefit was less pronounced in the ≥75 years age group, showing a negative correlation between age and OS [23]. Another multicenter retrospective study analyzing 928 elderly cancer patients aged ≥80 years receiving single-agent ICI therapy concluded that this population could benefit from ICI treatment with good tolerability, and the incidence of TRAEs did not differ significantly among patients aged <85, 85–89, and ≥90 years[24]. Subgroup analyses of the CheckMate series of trials revealed that elderly patients (≥75 years) treated with nivolumab demonstrated similar benefit trends in PFS and OS compared with their younger counterparts (<75 years), suggesting comparable efficacy [25].

Favorable clinical outcomes have been observed in patients with advanced refractory tumors based on the PRaG series of clinical studies conducted at our center. This protocol has been implemented in over one hundred hospitals in China, with more than five thousand patients having received PRaG therapy. The therapeutic rationale of PRaG therapy is that ionizing radiation exposes tumor antigens, creating an ‘*in situ* tumor vaccine effect.’ The PD-1 inhibitor and GM-CSF act as vaccine adjuvants, promoting the proliferation of antigen-presenting cells and enhancing the immune response efficacy of the tumor vaccine [26]. To better understand the efficacy and safety of PRaG therapy in the elderly, we retrospectively analyzed the outcomes of patients aged ≥75 years who received this treatment at our center.

Furthermore, evidence suggests that the phenomenon of age-related immune dysfunction (immunosenescence) may compromise the efficacy of single-agent immunotherapy in elderly patients. This provides a theoretical rationale for employing comprehensive combination strategies, such as the PRaG regimen, to potentially overcome immune resistance, although specific mechanistic markers were not evaluated in our current clinical cohort. The high-dose-per-fraction radiotherapy in the PRaG model is not intended for curative purposes but rather aims to promote the release of tumor antigens, thereby activating the body's antitumor immune response. Consequently, the treatment-related toxicity is low and can be tolerated by patients, offering a low-toxicity, effective treatment model for elderly cancer patients.

This study included 36 elderly patients who had received PRaG therapy over the past five years at our institution, with a median follow-up of 46.3 months. Among the 34 evaluable patients, efficacy assessment based on RECIST 1.1 criteria showed a CR rate of 5.6% (2 patients), a PR rate of 16.7% (6 patients), an SD rate of 44.4% (16 patients), and a PD rate of 27.8% (10 patients). The overall ORR was 23.5%, with a DCR of 70.6%. The mPFS was 7.0 months (95% CI: 2.4–11.6 months), and the mOS was 11.1 months (95% CI: 5.3–16.9 months). A multicenter retrospective study from Italy involving 86 patients aged ≥75 years with locally advanced or metastatic NSCLC (mean age 78.5 years, range 75–86) who received PD-1/PD-L1 inhibitors as first-line or subsequent therapy, reported that 69 patients (80.2%) had an ECOG performance status of 0 or 1. The overall mPFS was 5.6 months (1–36 months), and the mOS was 10.1 months (1.7–34.8 months). No significant survival difference was observed between patients younger than 80 and those older than 80 [27]. Compared with that study, slightly higher PFS and OS were observed in our cohort, even though 26.7% of patients in their study had PD-L1 ≥ 50% and 80.2% had a good performance status (ECOG 0 or 1), whereas in our study, 75% of patients had an ECOG score of ≥2. A real-world study from Japan enrolled 676 patients with advanced NSCLC receiving ICI monotherapy, including an elderly group of 137 patients (20.3%). The median age was 78 years (75–85) in the elderly group and 66 years (34–74) in the younger group. The mPFS (4.8 vs. 3.3 months, *P* = 0.1589) and mOS (12.3 vs. 13.0 months, *P* = 0.5587) were similar between the elderly and younger groups [28]. Compared with their findings, patients in our PRaG therapy group experienced greater PFS benefit. This could be related to the higher proportion of patients receiving ≥3 lines of therapy in their study, as well as the synergistic effect of radiotherapy and GM-CSF in the PRaG regimen, which stimulates the host's antitumor immune response.

In our study, 94.4% of patients experienced TRAEs of any grade. No grade 5 TRAEs were observed. Most adverse events (86.1%) were grade 1–2. Three patients (8.3%) experienced grade ≥3 TRAEs that led to treatment discontinuation: one case of immune-related myocarditis, one of immune-related dermatitis, and one of severe pneumonia. The incidence of grade 3–5 adverse events in this study was relatively low, although the overall incidence of adverse events was high. The most frequent events were fatigue, fever, and anorexia. Compared with ICI monotherapy, the incorporation of radiotherapy and subcutaneous GM-CSF injections in our study may have contributed to the higher incidence of these specific adverse events. However, no treatment-related deaths occurred, and the overall tolerability was good.

**Figure 3:**
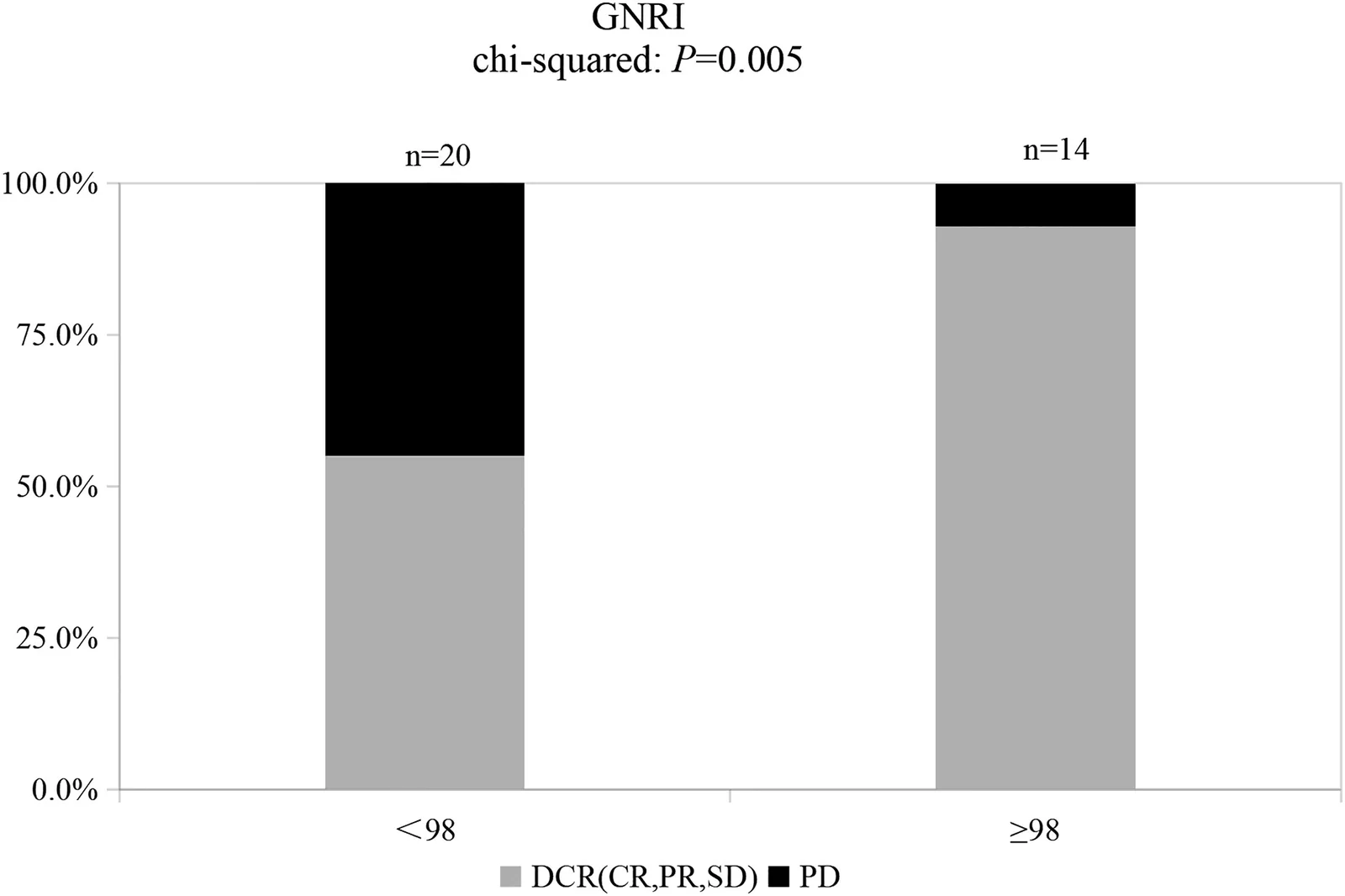
Disease control rates stratified by the established GNRI cutoff of 98. A statistically significant difference in DCR was observed between patients with GNRI<98 and those with GNRI ≥98 (*P* = 0.005 by chi-square test). CR, complete response; DCR, disease control rate; PD, progressive disease; PR, partial response; SD, stable disease.

**Figure 4:**
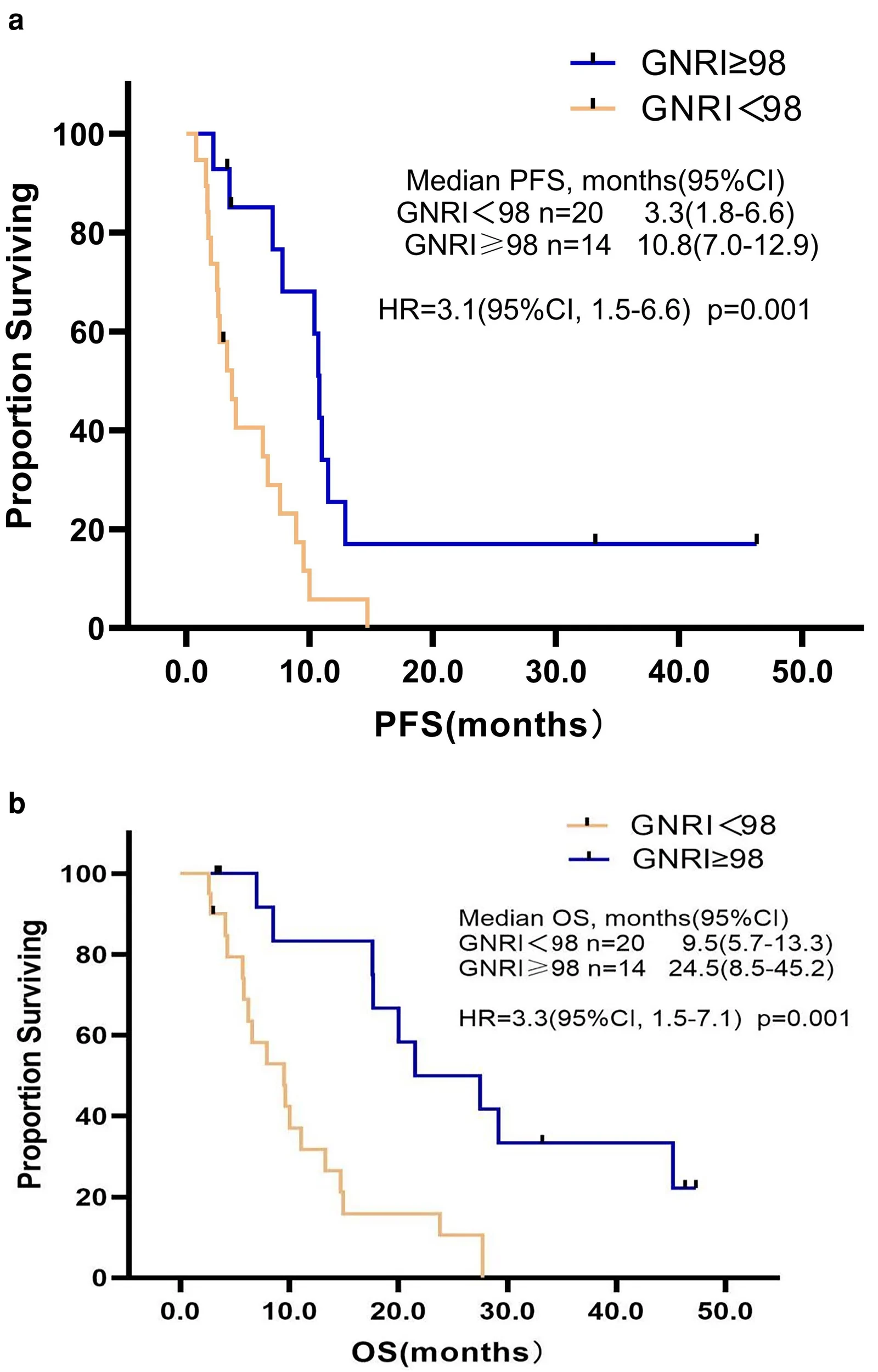
Kaplan-Meier curves for PFS (a) and overall survival (b) in elderly cancer patients treated with PRaG therapy, stratified by low versus high GNRI. Kaplan-Meier curves for PFS (a) and overall survival (b) in elderly cancer patients treated with PRaG therapy, stratified by the established GNRI cutoff of 98. Patients with higher baseline GNRI (≥98) demonstrated significantly longer PFS and OS compared with those in the lower GNRI (<98) group.

The population of elderly cancer patients is growing, and these individuals require complex, multi-layered care interventions to achieve optimal clinical outcomes from antitumor therapy. Among these, nutritional care is a critical yet often overlooked aspect. Malnutrition can severely impact the treatment outcomes of cancer patients [29] and is also associated with organ dysfunction, gut microbiota dysbiosis, metabolic abnormalities, and immune dysfunction [30]. Elderly cancer patients are often malnourished, and previous research has shown that malnutrition is a poor prognostic indicator for cancer patients receiving immunotherapy [31]. The GNRI, first proposed by Bouillanne *et al*. in 2005, is calculated based on the ratio of a patient's actual weight to their ideal body weight and their serum albumin concentration. Numerous clinical studies have demonstrated that GNRI is a simple and reliable prognostic indicator for various malignancies in the elderly, including esophageal and head and neck cancers, with lower values being associated with a higher risk of mortality [32, 33]. Poor nutritional status is a common comorbidity in patients with head and neck squamous cell carcinoma, with over 60% of patients being malnourished [34]. A retrospective study showed that among patients with recurrent or metastatic head and neck cancer receiving ICI therapy, a GNRI ≤ 98 was significantly associated with a lower DCR (*P* = 0.001), and a GNRI < 92 was an independent prognostic factor for PFS (*P* < 0.001) [35]. In a study by Peng *et al*., the prognostic value of GNRI scores in advanced NSCLC was analyzed, finding that GNRI was an independent prognostic factor for OS, regardless of age (<65 or ≥65 years). Additionally, hemoglobin was also identified as an independent prognostic factor for OS in these patients [36]. Consistent with these findings, our multivariate regression analysis identified baseline GNRI and baseline hemoglobin as independent prognostic factors for both PFS and OS in elderly patients receiving PRaG therapy. Furthermore, we observed significantly better objective responses in patients with a GNRI ≥ 98 than in those with a GNRI < 98 Figs 3 and 4.

The inflammatory response is intricately linked to tumor initiation and progression. Multiple studies have confirmed that the Neutrophil-to-Lymphocyte Ratio (NLR) is a predictive biomarker for patient prognosis in antitumor therapy [37–39]. In a retrospective study of 221 patients with advanced NSCLC (PD-L1 high expression ≥50%) receiving first-line pembrolizumab, a low derived NLR (dNLR < 2.6) was associated with a significantly higher ORR (52.4% vs. 24.7%, *P* < 0.001) and longer median PFS (10.4 vs. 3.4 months, *P* < 0.001) and OS (36.6 vs. 9.8 months, *P* < 0.001) compared with a high dNLR (≥2.6) [40]. That study further explored the potential mechanisms linking peripheral blood dNLR to immunotherapy efficacy, suggesting that an elevated dNLR is associated with reduced tumor immune infiltration. It has been previously confirmed that higher levels of CD8+ T cells in the NSCLC tumor microenvironment (TME) and increased PD-1 expression on these cells are positively correlated with efficacy following PD-1 inhibitor treatment [41]. The results of the PRaG 1.0 study suggested a correlation between the baseline CD4+/CD8+ ratio and post-treatment efficacy, and that an increase in peripheral blood CD3+ T cells, CD3+CD4+ T cells, CD3+CD8+ T cells, and NK cells after one cycle of treatment might be associated with better outcomes [1]. However, in the current study, we found no correlation between lymphocyte subset counts or fine-grained lymphocyte phenotyping and treatment efficacy. Nevertheless, the baseline neutrophil-to-CD8+ T lymphocyte ratio was identified as an independent prognostic factor for OS (HR = 0.95, 95% CI: 0.89–0.99, *P* = 0.044). We will conduct further investigation into the immune status of elderly cancer patients and the identification of immune-related biomarkers associated with PRaG therapy efficacy in our prospective clinical trial (NCT06112041).

Patients with liver metastases historically exhibit poorer responses to immunotherapy due to the uniquely immunosuppressive TME of the liver [42]. In our study, the presence of liver metastases was associated with treatment efficacy and was an independent prognostic factor for PFS. While our study included a small subset of patients with liver metastases (*n* = 10), the limited sample size precludes any definitive clinical or mechanistic conclusions regarding the efficacy of the PRaG regimen in this specific subgroup, warranting further investigation in larger cohorts.

For contextual interpretation, our previously published PRaG 1.0 study enrolled 54 patients, including 25 males and 29 females, with a median age of 60 years (range, 31–77 years), of whom 7 were aged ≥75 years. In the intention-to-treat population, the ORR was 16.7%, the DCR was 46.3%, the median PFS was 4.0 months (95% CI, 3.3–4.8 months), and the median OS was 10.5 months (95% CI, 8.7–12.2 months) [1]. Compared with these previously reported results, the elderly cohort in the present study showed numerically favorable efficacy outcomes. However, this comparison should be interpreted with caution, as the two cohorts differed in study design, baseline characteristics, and treatment exposure. We hypothesized that the inclusion of a 25% treatment-naïve fraction contributed to the improved outcomes observed in our study compared with the historical PRaG 1.0 cohort. This plausible explanation is now supported by our exploratory subgroup analysis, which revealed that treatment-naïve patients achieved substantially longer median PFS (8.93 vs. 3.67 months) and OS (17.73 vs. 9.63 months) than pretreated patients within the same cohort. While limited statistical power precluded significant *P*-values, this distinct numerical advantage underscores the potential benefit of introducing the PRaG regimen in earlier lines of therapy.

Several limitations to the present study must be acknowledged. First, this is a single-center retrospective analysis with a small sample size (*n* = 36) and heterogeneous tumor types. There is inherent selection bias, as the administration of the PRaG regimen in this elderly, poor-ECOG cohort heavily relied on physician judgment. Consequently, this cohort may be enriched with patients pre-selected for relative fitness, limiting the generalizability of our findings to the broader, unselected elderly population. Furthermore, the lack of a younger comparator group (<75 years) restricts our ability to definitively compare the magnitude of benefit and toxicity across age ranges, rendering our findings descriptive and exploratory.

Second, the retrospective design precluded the collection of comprehensive clinical and biological metrics. From a geriatric perspective, while we evaluated the aCCI and GNRI, we lacked a full Comprehensive Geriatric Assessment (CGA), frailty screening (e.g. G8), and Patient-Reported Outcomes (PROs/QoL). Biologically, the lack of centralized testing and the high proportion of missing PD-L1 data (69.5%) severely precluded any meaningful biomarker correlative analysis.

Third, statistical constraints exist due to the exploratory nature of this study. The limited number of clinical events restricted our survival analyses; although we rigorously adhered to the events-per-variable (EPV) rule in Cox models to minimize overfitting, residual confounding may still exist. Additionally, without formal adjustments for multiple comparisons, borderline-significant findings should be interpreted with caution as potential false positives.

Finally, while we applied a previously validated literature cutoff (GNRI = 98) to prevent circular reasoning, its precise prognostic value for the PRaG regimen requires prospective validation. To address these limitations, a prospective trial (NCT06112041) is currently underway to further validate this regimen in older adults, explicitly incorporating complete CGA, G8 screening, and robust QoL evaluations.

## Conclusion

In conclusion, our retrospective analysis suggests that the PRaG regimen appears feasible and tolerable in selected elderly patients (≥75 years) with advanced solid tumors treated at our center. However, given the inherent limitations of a small, single-center retrospective series lacking a younger comparator group, these findings are strictly descriptive and do not support an immediate recommendation for first-line clinical use. Prospective validation, such as the ongoing clinical trial (NCT06112041), is essential before any practice-changing conclusions can be drawn. Furthermore, we hypothesize that combining the PRaG regimen with robust nutritional support may further optimize outcomes in this vulnerable population; however, this remains an exploratory hypothesis requiring direct evaluation in future prospective studies.

## Data Availability

The data supporting the findings of this study are available from the corresponding author upon reasonable request.
